# Intracranial Calcification and Seizure with Down Syndrome: A Case Report

**DOI:** 10.31729/jnma.7950

**Published:** 2022-12-31

**Authors:** Nilshan Rai, Monika Thapa, Merina Pokharel, Jinee Acharya, Dhirendra Yadav

**Affiliations:** 1 KIST Medical College and Teaching Hospital, Gwarko, Lalitpur, Nepal; 2 Department of Neurology, National Neuro Center, Maharajgunj, Kathmandu, Nepal; 3 Patan Academy of Health Sciences, Lagankhel, Lalitpur, Nepal

**Keywords:** *basal ganglia*, *Down syndrome*, *seizure*, *trisomy 21*

## Abstract

Down syndrome is a genetic disorder caused by an extra copy of chromosome number 21. New onset of seizure in adults with Down syndrome is rare. The exact pathogenesis of intracranial calcification and seizure in Down syndrome is unknown, however, a possible association between hypocalcemia and vitamin D deficiency in Down syndrome was reported. An 18-year-old girl with nasal bridge, mongoloid slants, clinodactyly and saddle gap of toes, and prominent Downs phenotypes was present with a low level of parathyroid hormone, calcium, and vitamin D. Due to a higher prevalence of intracranial calcification in people with Down syndrome, there is an increased possibility of hypocalcemia and vitamin D deficiency. Hence, serum levels of calcium and vitamin D should always be checked before starting treatment with anti-epileptic drugs.

## INTRODUCTION

Down syndrome (DS) is a genetic disorder caused by an extra copy of chromosome 21. Patients with down syndrome have a higher incidence of around 8% of seizures than the general population.^1^ Down syndrome is not identified as a common cause of intracranial calcification but even it is a common finding in computed tomography's average range of 10.7-26.7%.^2^ Intracranial calcification and seizure in DS are rarely reported. Our patient has calcification along with seizure which could have been a result of a link between hypocalcemia and vitamin D deficiency but the exact mechanism was unknown.

## CASE REPORT

A 18-year-old girl with a wide nasal bridge, mongoloid slants, clinodactyly and saddle gap of toes, and prominent features of Down syndrome, presented to the Neurology Outpatient Department with a history of multiple episodes of seizures in a cluster. The episodes of seizure started when she was at the age of 7 months old and has been under regular medication (Epilex Chrono 500 mg once a day). Her history of seizures was suggestive of focal onset with secondary generalization followed by postictal loss of consciousness. Natal history was significant for low birth weight (1600 gm) followed by excessive sleep, hypotonia, and delayed developmental milestones. She was diagnosed and proven to have trisomy 21 at the age of 13 months. She has a history of intellectual disability, phenotype of Down syndrome, and hypothyroidism and no other physical abnormality could be detected on physical examination.

**Figure 1:**
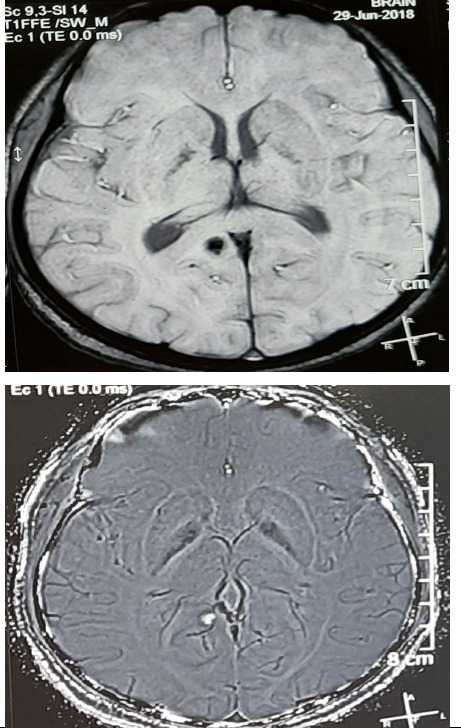
MRI shows calcification in the brain.

However, the cardiovascular, respiratory and abdominal examinations of the patient were normal. Her complete blood count, random blood sugar, magnesium, and renal and liver function tests were within normal limits but low calcium (6.8 mg/dL; normal 8.5-10.2 mg/dL), vitamin D (15.3 ng/ml; normal range 20-40 ng/ml). There is no radiological or clinical finding of rickets. However, her lipid profile was slightly above the upper limit and uric acid was raised (10.5 mg/dl). Her thyroid function tests were normal with appropriate treatment. Her echocardiogram, electrocardiogram and ultrasonography of the abdomen and pelvis showed no abnormality except for Grade I fatty liver. Her electroencephalogram revealed sharpish discharges with theta to delta waves. Her magnetic resonance imaging (MRI) scan of the head showed multiple intracranial calcifications (Figure 1).

There was no other significant medical or family history. She was started on oral calcium and calcitriol supplementation and maintained dietary habits. The diagnosis of Down syndrome with seizures caused is unknown with intracranial calcification. There was control of the seizures and now she is under regular follow-up.

## DISCUSSION

Down syndrome patients with seizure and intracranial calcification were rare presentations. Patients with DS have a greater incidence of seizures (febrile and afebrile) than the generalized population. According to a study, seizures in DS were bimodal distribution with a 40% chance to develop before 1 year and 40-50% develop after the age of 30 years, thus making the new onset of seizures in adolescents with DS rare.^2^ There is no unanimity that Down syndrome is a frequent cause of intracranial calcification.^3^ Calcification most commonly involves basal ganglia which are similarly seen in our case. Few cases show neurological symptoms have been reported but in general, there is no sign or symptoms attributable to calcification which is a similar presentation in this case report.

The exact pathogenesis of calcification in Down syndrome is unknown but around 10.7-26.7% of calcification findings were found on a head CT scan of Down syndrome.^3^ Seizures present in infants may be due to genetic structural brain abnormalities, abnormal cortical lesions, underdeveloped synaptic profiles, etc. but the mechanism of seizure in adults has not yet been explained completely in DS.^4^ The prevalence of vitamin D deficiency has more common in children with DS but rare in adult^5,6^ with DS which is similar to this case report she has a low vitamin D level. As such DS is not commonly considered one of the major causes of intracranial calcification but MRI detects intracranial calcification in Down syndrome. Sometimes hypothyroidism can predispose to intracranial calcification mainly in the basal ganglia by deposition of calcium crystals in cerebral tissue in the brain due to decreased calcium level (hypocalcemia).^2^ However, the exact pathogenesis of intracranial calcification in basal ganglia in DS is not clear, similar to this case report. That link between hypoparathyroidism, hypocalcemia and vitamin D deficiency and seizures in DS is being reported in this literature, to our best knowledge.

This case demonstrates how crucial it is to indicate hypocalcemia even in adolescents with DS who are having seizures and to have serum ionised calcium levels checked before beginning the antiepileptic medication. Hypocalcemia causes seizures because the mechanism of neuronal excitability increases due to a lessen in extracellular calcium concentration compared to an intracellular level that causes a seizure. As a potential pathogenetic reason for the higher prevalence of intracranial calcification and seizures in people with DS, this case also increases the probability of clinical/subclinical hypocalcemia and vitamin D deficiency.
